# Recognition of Reovirus RNAs by the Innate Immune System

**DOI:** 10.3390/v12060667

**Published:** 2020-06-20

**Authors:** Andrew T. Abad, Pranav Danthi

**Affiliations:** Department of Biology, Indiana University, Bloomington, IN 47405, USA; atabad@iu.edu

**Keywords:** reovirus, innate immunity, sensing, dsRNA, interferon

## Abstract

Mammalian orthoreovirus (reovirus) is a dsRNA virus, which has long been used as a model system to study host–virus interactions. One of the earliest interactions during virus infection is the detection of the viral genomic material, and the consequent induction of an interferon (IFN) based antiviral response. Similar to the replication of related dsRNA viruses, the genomic material of reovirus is thought to remain protected by viral structural proteins throughout infection. Thus, how innate immune sensor proteins gain access to the viral genomic material, is incompletely understood. This review summarizes currently known information about the innate immune recognition of the reovirus genomic material. Using this information, we propose hypotheses about host detection of reovirus.

## 1. Introduction

Viral infections represent a molecular arms race, where viruses must overcome host defenses in order to replicate. Target hosts have evolved complex strategies to fight off viruses. Many of these strategies require the infected cells to be able to detect that a viral infection has begun. The major sensing mechanism to recognize viral infections, involves the detection of viral nucleic acids through endosomal and cytoplasmic sensor proteins. Being able to distinguish between self and viral nucleic acids is not trivial, as viruses have evolved ways of cloaking their nucleic acids to appear similar to their hosts. When a host is successful at sensing viral nucleic acid, it begins a signaling cascade to establish an antiviral state, through the activation of transcription factors.

**Figure 1:**
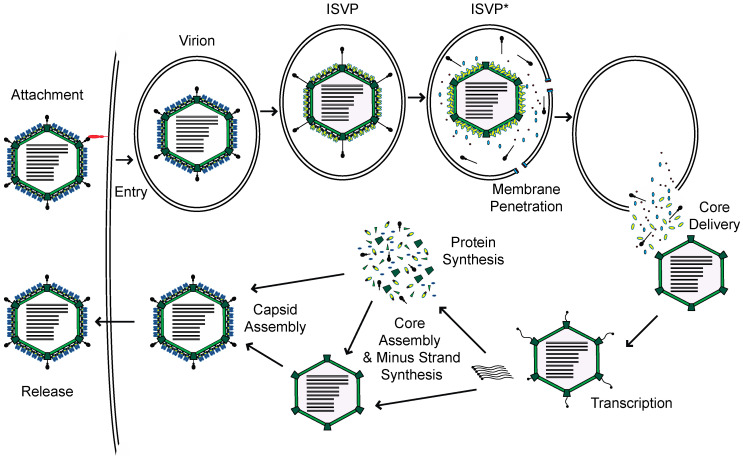
Reovirus replication cycle.

Mammalian orthoreovirus, henceforth reovirus, is a member of the *Reoviridae* family of viruses. Reovirus is comprised of two concentric protein shells which encapsidate ten segments of dsRNA genome [1]. Reovirus enters host cells through receptor-mediated endocytosis (Figure 1) [2]. Within endosomes, the outer capsid is proteolytically digested by host acid-dependent cathepsin proteases, to generate intermediates called infectious subvirion particles (ISVPs). This digestion and subsequent conformational transition to form ISVPs, releases small peptides from the outer capsid, which form pores in the endosome and allow for the deposition of the viral core into the cytoplasm. Within the cytoplasm, the core is transcriptionally active. The encapsidated RNA-dependent RNA polymerases use each genome segment as a template to produce mRNAs [3]. These mRNAs, which are capped by the viral capping enzymes also present in the particle, are extruded through turrets in the core into the cytoplasm, for translation by host ribosomes. Once viral proteins are generated, progeny cores can form with viral plus-strand RNA, which transcribe new minus-strand RNA. These newly formed cores can undergo secondary rounds of transcription, or acquire outer capsid proteins for eventual release from the cell [1].

Reovirus infection leads to induction of type I and type III interferon (IFN) responses in cell culture and mouse models [4,5,6,7,8,9,10,11,12,13]. The type III response appears to be important in limiting reovirus replication in intestinal epithelial cells [9,12]. However, the mechanism by which the type III response is activated following reovirus infection, has been minimally explored. Thus, this review will focus only on the type I IFN response. The type I IFN response is capable of restricting reovirus replication in cell culture and providing protection in infected mice [5,6,7,14]. The antiviral response is initiated following the sensing of nucleic acids by activation of the transcription factors—Interferon Regulatory Factor 3 (IRF-3) and Nuclear Factor kappa-light-chain-enhancer of activated B cells (NFκB) [15]. IRF-3 remains inactive in the cytoplasm until it is phosphorylated by TANK Binding Kinase 1 (TBK1) and IκB kinase epsilon (IKKε) [15]. Once phosphorylated, IRF-3 translocates to the nucleus to initiate transcription of type I IFN. These IFN proteins are secreted to signal through the IFN-α/βreceptor (IFNAR) on the cell surface in an autocrine and paracrine manner, for the induction of interferon stimulated genes (ISGs), which largely act to generate an antiviral state, consequently rendering cells more resistant to infection [16]. Similarly, NFκB is activated during reovirus infection [17]. NFκB is sequestered in the cytoplasm by the Inhibitor of κB (IκB) complex [15]. Inhibition of NFκB is released when the upstream activating IKK complex phosphorylates, IκB and NFκB. The phosphorylation of IκB leads to its degradation, while NFκB is released, enabling it to translocate to the nucleus to initiate transcription. In some cell types, NFκB cooperates with IRF-3 to induce IFN expression. In addition, both IRF-3 and NFκB can drive the expression of target genes, which contributes to the dampening of viral replication, independent from IFN [15]. The antiviral response generated from IFN signaling results in the activation of various proteins, whose function is to dampen viral replication [16]. Such proteins include Protein Kinase R (PKR), which leads to the inhibition of RNA translation following activation; Oligoadenylate Synthetase (OAS), which helps activate RNAse L to degrade RNA; and Adenosine Deaminase (ADAR1), which can edit RNAs, causing mutations in viral genomes; among others [16]. Information about the antiviral ISGs induced in response to reovirus infection, and the reovirus proteins involved in inhibiting this response, has recently been compiled [18].

In this review, we summarize the role of various sensors of reovirus infection. We also compile currently available information about the timing of viral RNA detection during reovirus infection.

## 2. Sensors of Reovirus

Mammalian cells have evolved multiple types of sensor proteins, which are triggered during various types of infection, in response to foreign materials [15]. These sensors are largely known as pattern recognition receptors (PRRs) and interact with pathogen-associated molecular patterns (PAMPs), which can typically only be molecules generated by pathogens, such as certain proteins, nucleic acids, or sugars. For viral infections, the detection of nucleic acid, which contains features that are not normally present in the cell, or the presence of nucleic acid in subcellular compartments where it is not present in a physiological context, acts as a powerful stimulus for generating an innate immune response.

Retinoic acid-inducible gene-I (RIG-I) and melanoma differentiation-associated 5 (MDA-5), are cytoplasmic sensors of uncapped RNA or long dsRNA, which are a part of the RIG-I like receptor (RLR) family. Upon interaction with their ligand, these sensors stimulate the activation of the adaptor protein—mitochondrial antiviral-signaling (MAVS)—which leads to the downstream activation of IRF-3 and NFκB. RIG-I^−/−^ or MDA-5^−/−^ mouse embryonic fibroblasts (MEFs), show decreased ISG expression following reovirus infection compared to wildtype cells, but the IFN response is not completely lost in the absence of either receptor [19]. However, the adaptor protein MAVS, which functions downstream of both RIG-I and MDA-5, is essential for generating an IFN-based innate immune response to reovirus infection, in both primary and transformed cell types [19,20,21,22,23]. These data indicate that RIG-I and MDA5 are likely to have a compensatory and somewhat redundant role in the sensing of reovirus infection. In HEK293T (human embryonic kidney) cells infected with reovirus, an overexpression of dominant negative RIG-I in reovirus infected cells, leads to a reduced ability to activate an IRF-3/7 reporter [21]. Why MDA-5 does not compensate for the absence of a functional RIG-I in these cells is unclear, but we speculate that this may be related to the relative levels of these two sensors in different cell types. Knockout of both RLRs allows for an increased replication of reovirus [22]. Consistent with this, reovirus replication in the intestine and lymph nodes of MAVS^−/−^ mice is enhanced [22]. Thus, the RLR-MAVS pathway appears key to the mounting of an innate immune response to reovirus in vivo.

While RIG-I and MDA-5 appear to be the major sensors of reovirus infection, other helicases have been shown to contribute to the innate immune response in some cell types of the immune system. In myeloid dendritic cells (mDCs), both stable and transient knockdown of DexH-Box Helicase 9 (DHX9), leads to the decreased ability of these cells to secrete type I IFN following reovirus infection [24]. DHX9 is capable of responding to both short and long stretches of the dsRNA mimic, polyI:C. MAVS also interacts directly with DHX9 through its caspase activation and recruitment domain (CARD), and both DHX9 and MAVS are necessary for a robust activation of IRF-3 and NFκB. Similarly, the helicase complex of DEAD-Box Helicase 1 (DDX1), DDX21, and DHX36 are capable of recognizing dsRNA and stimulating an antiviral response through a separate adaptor molecule TRIF (TIR-domain-containing adapter-inducing interferon-β), in mDCs [25]. Knockdown of each of the complex components leads to a reduced capacity to secrete cytokines following reovirus infection. Knockdown of the helicase complex components or TRIF also leads to the reduced capacity of these cells to stimulate the activation of IRF-3 and NFκB. The contribution of these non-RLR sensors to the innate immune response to reovirus in other cell types or in infected animals, has not been evaluated.

Toll-Like Receptor 3 (TLR3) is a dsRNA sensor, which localizes to endosomes [15]. The interaction of TLR3 with its ligand allows signaling through TRIF, to promote the downstream activation of IRF-3 and NFκB. TLR7 and TLR8 are known to recognize ssRNA in the endosome and signal via MyD88, to activate the same response. Although conflicting evidence exists regarding the role of TLR3 in sensing reovirus infection in cultured cells [20,23,26,27], TLR3 does not appear to regulate reovirus pathogenesis in the CNS, and is not important for the clearing of reovirus infections [28,29]. The genetic deletion of TLR3 does not mimic the uncontrolled replication of reovirus observed in IRF-3 or IFNAR^−/−^ animals [4,29,30]. Viral titers from TLR3^−/−^ or MyD88^−/−^ mouse organs are similar to titers from WT mouse organs. Correspondingly, the absence of TLR3 does not enhance viral pathogenesis [28,29]. Similarly, MyD88^−/−^ mice do not succumb to reovirus infection. These data indicate that the RNA sensing TLRs do not play a significant role in sensing and responding to virus infection.

## 3. Ligands

Reovirus genomic RNA has frequently been used as a potent stimulator of the innate immune system [20,22,23,31,32]. The 5′-end of the plus strand is capped, while the 5′-end of the minus strand has a free diphosphate [33,34,35,36]. The incubation of L929 cells with purified reovirus genomic dsRNA mixed with DEAE-dextran, which likely results in its delivery into cells, induces a robust IFN response [31]. The different size classes of reovirus genome segments differ in their ability to interact with RIG-I and MDA-5, indicating that these sensors have a preference for particular sizes of dsRNA [22,32]. The small (S) segments range in size from 1.2 to 1.4 kb, the medium (M) from 2.2 to 2.3 kb, and the large (L) from ~3.9 kb [1]. Transfection with either the S, M, or L RNA segments, stimulates an IFN response [22,32]. RIG-I^−/−^ cells are unable to induce IFN following transfection of the S genomic segments, but induce reduced amounts of IFN in response to either the M or L segments. In contrast, MDA-5^−/−^ cells are fully capable of inducing IFN in response to the S segments, but have a diminished response to both the M and L segments [32]. Small, abortive transcripts of ~2–9 nts can also be isolated from reovirus particles [37], but these oligonucleotides are unable to induce an IFN-β reporter [22]. These data indicate that all three lengths of reovirus genome segments are potent ligands for these RLRs—where RIG-I primarily senses smaller RNA segments, MDA-5 recognizes longer dsRNAs. We expect that the genomic dsRNA of each segment is therefore capable of stimulating an innate immune response. These data may also explain why RIG-I and MDA5 play a redundant role in inducing type I IFN expression in infected cells. The capacity of the reovirus genome to elicit an IFN response, depends on phosphate groups present on the 5′-end of the minus strand [22]. Calf intestinal alkaline phosphatase (CIP) treatment on reovirus genomic RNA, removes free phosphate groups. CIP-treated reovirus RNA demonstrates a drastically reduced ability to stimulate an IFN-β reporter, and to induce IFN-β mRNA [20,22]. 

In the context of infection, the incoming genomic dsRNA of reovirus appears to be the most likely candidate for recognition by cytoplasmically localized RLRs, to induce an antiviral response. Blocking viral transcription and dsRNA synthesis does not block IFN induction [20,23]. In addition, a UV-inactivated virus stimulates an IFN response during infection, but is unable to replicate [38]. Similarly, infection of chicken cells which are non-permissive to reovirus, leads to the release of IFN, even though the virus fails to transcribe mRNA [39]. Early events in the cell entry of reovirus are required for IFN induction. For example, blocking early entry steps of reovirus leads to a reduced immune response [20,23]. Inhibiting endosomal acidification with ammonium chloride (AC), decreases the IFN-β mRNA made following reovirus infection [20]. Inhibiting endosomal proteases with E64 or AC treatment also reduces reovirus-induced IRF-3 activation, indicating that virus disassembly is required for the induction of the innate immune response [21,23]. In addition, empty reovirus particles lack the genome complete cell attachment and disassembly, but do not induce IFN secretion or IRF-3/7 activation [21,38]. Thus, the capsid proteins and their disassembly is not sufficient to induce an innate immune response. These data also indicate that the incoming genomic dsRNA is required for the activation of the innate immune response. However, the dsRNA is thought never to be exposed to the host cytoplasm [40,41]. Thus, how the RLRs gain access to incoming genomic dsRNA in an infected cell, is undetermined.

The entry pathway of reovirus into cells may affect the induction of the innate immune response to infection. Reovirus virions undergo a disassembly step to shed their outer capsid—to facilitate membrane penetration and deposition of the core into the cytoplasm. During this disassembly, the reovirus transitions to form ISVPs. ISVPs are infectious particles, which can also be generated in vitro or in mouse intestines [42,43,44]. ISVPs induce little to no immune response following infection [27,45]. The reason for this difference between virions and ISVPs is not currently known, but could be due to differences in how the two types of particles enter the cell. While both virions and ISVPs utilize the same receptor for binding, virions must reach the late endosomal compartment, where acid-dependent cathepsin proteases catalyze the necessary maturation steps for entry into the cytoplasm [46,47,48]. Conversely, since ISVPs are already disassembled, they are not dependent on endosomal proteases and can enter cells directly at the plasma membrane, or early in the endocytic sorting pathway [49,50,51]. Though there is a correlation between the uptake mechanism and the innate immune response, whether this relates to differential detection of virions and ISVP is yet unknown. It is also possible that ISVPs activate a pathway that suppresses innate immune signaling [45]. 

## 4. Sensing of Other dsRNA Viruses

The sensing mechanism of reovirus appears similar to that reported for other well-studied dsRNA viruses that infect vertebrates. These similarities are highlighted below.

Rotavirus is another member of the *Reoviridae* family of viruses, containing 12 segments of dsRNA genome. Similar to reovirus, rotavirus infection leads to the induction of IFN [52,53,54,55,56]. This IFN induction requires the RIG-I/MDA5/MAVS signaling pathway, both in cell culture [53,54,55] and in mouse models [54,55]. TLR3 does also not appear to be important in restricting rotavirus infection [54]. As the same sensors are involved in sensing rotavirus and reovirus, it is expected that the dsRNA genome is the likely PAMP. Much like reovirus, the rotavirus genome is also protected by core proteins throughout infection [57,58]. A signal for dsRNA has been found in the cytoplasm of rotavirus infected cells, but it has been suggested that this could be viral mRNA with a double-stranded secondary structure [59]. The inefficient capping of viral mRNA has also been proposed as a mechanism for IFN induction, as this could lead to the presence of plus-strand RNA, with a free di- or triphosphate in the cytoplasm to be sensed [56]. Infecting cells with inactivated, non-infectious rotavirus particles still leads to IFN induction [60,61,62,63]. These data indicate that the incoming genome is likely the PAMP for IFN induction during rotavirus infection. The innate immune response to rotavirus infection has been reviewed previously [64].

Avian reovirus shares many similarities with mammalian reovirus, with both forming double-layered particles containing 10 segments of the dsRNA genome [65]. While avian reovirus is resistant to the antiviral effects of an ISG, PKR [66,67], IFN is still induced during infection of chicken embryo fibroblasts. The induction of IFN in these cells is not dependent on replication, as neither UV-inactivating the virus or blocking viral transcription with ribavirin, block IFN production [68]. Neither the stimulating ligand nor the sensor have been identified for IFN induction during avian reovirus infection, but the incoming dsRNA genome is a likely candidate, as replication is not required for IFN induction [68]. Similarly, sensors of dsRNA are likely to be important in the sensing of avian reovirus. While chicken cells do not encode an ortholog of mammalian RIG-I, orthologs of MDA5/MAVS and TLR3 could be important in this system [69].

Bluetongue virus, another member of the *Reoviridae*, is an arbovirus which primarily infects ruminants. Bluetongue virus induces IFN during infection in vitro [70] and in vivo [70,71]. As with reovirus, RIG-I, MDA5, and MAVS are important in inducing IFN in epithelial cells [72]. Furthermore, in plasmacytoid dendritic cells (pDCs), it has been suggested that IFN expression occurs through the adaptor MyD88 [73], which is downstream of most TLRs [15]. In pDCs, UV-treated bluetongue virus induces IFN, indicating that viral replication is not needed for sensing in this system [73]. Additionally, blocking endosomal maturation also blocks the IFN induction caused by UV-treated bluetongue virus infection [73]. As with other members of the *Reoviridae*, the bluetongue virus dsRNA genome can function as a stimulus for IFN induction [70]. Replication of the bluetongue virus appears to be necessary for IFN induction in some systems [72], but not required for others [74]. A more detailed review of the IFN response to bluetongue virus has been published [75].

## 5. Conclusions

**Figure 2:**
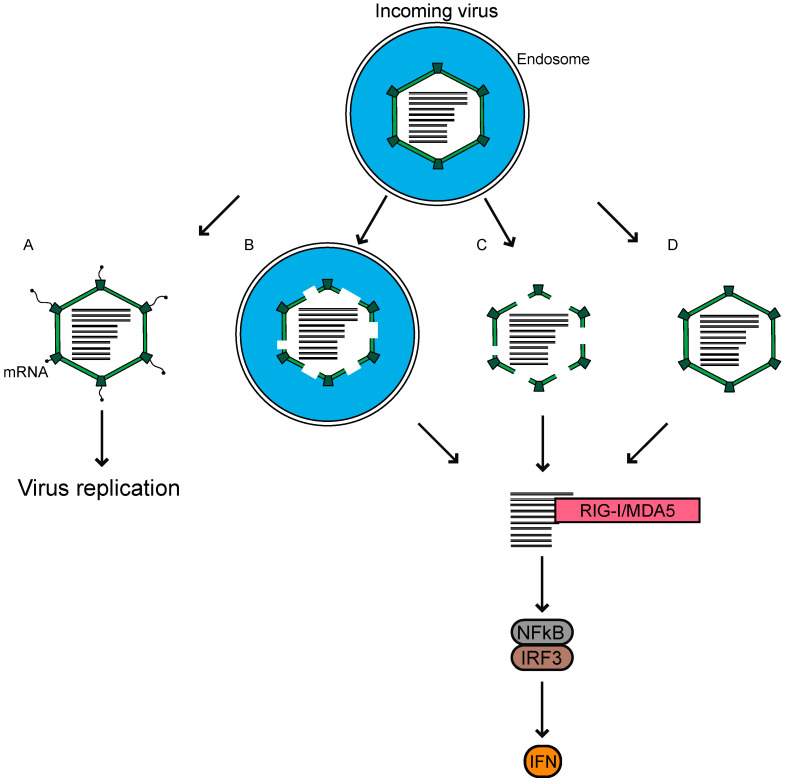
Reovirus virions enter host cells through receptor-mediated endocytosis. We propose potential fates for incoming virions that reach host endosomes. (**A**) Particles undergo disassembly within endosomes, which mediates pore formation in the endosome for the deposition of transcriptionally active cores to initiate replication. (**B**) A proportion of the particles may be unable to exit endosomes properly, where they are degraded to release genomic RNA. (**C**) Partially degraded particles enter the cytoplasm where they fall apart. (**D**) Intact cores are deposited into the cytoplasm, but later fall apart or are degraded by the host. In B–D, reovirus genomic RNA is made available to the host for sensing by RIG-I-like receptors (RLRs) and the subsequent induction of infectious subvirion particles (ISVP). Each of these possibilities could happen within the same cell, such that the virus can both establish infection and be sensed.

For reovirus infection, the major players contributing to an antiviral response have been identified. The incoming genome of reovirus is sensed primarily through the cytoplasmic RLRs for the MAVS-dependent activation of transcription factors, and the induction of IFN leading to a reduction in viral replication. A major remaining question in the field, is how host sensors gain access to the viral genome, which remains protected by the core throughout infection. Reovirus preparations have a particle-to-PFU ratio of ~100:1 [1]. Neither the basis for this ratio, nor the fate of the remainder of non-infectious particles within the cells, is known. We propose three possible ways in which these non-infectious viruses may be the source of dsRNA, which triggers the innate immune response (Figure 2). First, when the infection is initiated using virions, a proportion of the internalized virions remain associated with endosomes/lysosomes, even later in the infection [76,77]. These particles, which fail to escape the endosome or lysosome, may not launch the infection, but are degraded over time and release the dsRNA genome. The viral genome from within endosomes may reach the cytoplasm for detection, through pores formed either by the virus or by another mechanism. Secondly, it is possible that a proportion of the cores delivered into the cytoplasm are not intact following their passage through the endosomes, and cannot initiate RNA synthesis to launch the infection. Instead, their dsRNA is detected by RLRs to initiate innate immune signaling. Thirdly, the cores could be delivered intact into the cytoplasm, but some proportion of the cores are broken down over time or rendered permeable by cellular machinery, to release or expose dsRNA for RLR detection. We think that investigations into these, alongside other possible mechanisms, could explain how the reovirus dsRNA genome from entering particles, induces an innate immune response. Understanding the basis of why virions and ISVPs differ in their capacity to cause an innate immune response is another significant area of interest. A potential link between entry-related events and the capacity of host cells to respond to infection, will be useful for further understanding the complex interactions between the host and the virus, which is built to shield its genomic material from detection by the host.
